# Parturient Paralysis

**Published:** 1903-04

**Authors:** D. R. Kohler


### PARTURIENT PARALYSIS.

Dr. D. R. Kohler, Boyertown, Pa., in his paper before the Schuylkill Valley Veterinary Association, December, 1902, referring to the subject of parturient paralysis, attributes the frequency of the disease in his district to the high feeding of animals kept for milking purposes only. The author noted the fact of the disease rarely occurring after the first calving, but nearly always after the birth of the third or fourth calf, and usually when they are in the best of condition. After describing the group of nervous symptoms the animals display at the onset of the disease, he also referred to the many deaths from inflammation of the bowels and a general discrasia of the intestinal tract.

Referring to the treatment, he considered that animals which, by virtue of their general condition, would be prone to the disease, should have plenty of exercise, little or no grain, laxative food and salines, and prompt removal of the placenta when retained.

Sulphate of strychnine, 2 gr.; Barbadoes aloes, ½ oz.; nitrous ether, ½ oz.; aromatic spirits of ammonia, ½ oz., hourly; then every two hours till signs of recovery; then every three hours, given with a small vial, and Schmidt's iodide of potassium treatment of the mammary glands, and the general management of such cases, of frequent turning and keeping the animals well propped up, had given him the best results in the treatment of this disease.

---

Surgical Treatment of Splints. A horse, five years old, presented at the supra-internal part of the left canon a hard splint as large as an egg—6 cm. long and 3½ cm. wide. The splint caused no lameness, but was merely a blemish. The seat of operation had previously been shaved, disinfected, and an antiseptic dressing applied. After casting the horse and applying an Esmarch bandage the operation was performed as follows: A crucial incision 10 cm. long and 8 cm. wide over the tumor and the flaps dissected away; a crucial incision of the periosteum (which was raised) to the surrounding healthy bone. The tumor was then chiselled off to the normal level of the bone and smoothened with a curette, the periosteum and the skin sutured separately, and the parts dressed aseptically.

In fifteen days the dressing was removed. There was no trace of suppuration. At the seat of operation there was a small enlargement, which afterward disappeared. The horse returned to work in a month. A cicatrix and a scarcely perceptible swelling are the only indications of the splint.—*Zeitschrift f. Veterinarkunde.*

